# Mercurius-vivus

**Published:** 1896-08

**Authors:** 


### HOMŒOPATHIC PHYSICIAN,

A MONTHLY JOURNAL OF

HOMŒOPATHIC MATERIA MEDICA AND CLINICAL MEDICINE.

"If our school ever gives up the strict inductive method of Hahnemann, we are lost, and deserve only to be mentioned as a caricature in the history of medicine."—CONSTANTINE HERING.

Vol. XVI. AUGUST, 1896. No. 8.

### EDITORIAL.

MERCURIUS-VIVUS.—Continuing these notes, there is erysipelatous inflammation extending from the back around the abdomen like a girdle. It resembles herpes zoster, and causes us to think of three principal remedies—Mercurius-vivus, Rhus-tox., and Apis.

The twitching of the arms and fingers occurring under Mercury reminds us also of Ignatia. When we see trembling of the hands and fingers in brandy drinkers, the patient is unable to hold anything in his hand until he has had a glass of brandy, we should think of Mercurius. Such patients are apt to have chronic diarrhœa, and if we examine the tongue we will frequently find imprints of the teeth along its edges. Sepia is similar to Mercurius in having moist itch on the hands, with violent itching at night.

Contraction of fingers indicates not only Mercurius, but Arsenicum and Causticum. Swelling of the finger joints indicates Mercurius and Calcarea-carbonica. Mercurius is an important remedy in hip-joint disease. Mercurius has cold sweat on the forehead and on the feet. The cold sweat on the forehead reminds us of Veratrum, of which it is the key-note. Under Mercurius the bone pains are worse at night.

Painful swelling of metatarsal bones indicates Mercurius and Asafœtida. Mercurius has rheumatic and arthritic pains, worse at night and attended with profuse perspiration, which gives no relief.

This symptom, as stated, gives three key-notes of Mercurius:

(1). Aggravation at night.

(2). Rheumatic pains, attended with much perspiration.

(3). Perspiration gives no relief.

The perspiration is apt to be cold, and is usually oily. This, too, is a key-note.

We have here suggested to us the following comparisons:

Hepar, sweat without relief.

Hellebore, cold sweat without relief, followed by paralysis in cases of myelitis.

Chelidonium, perspiration without relief in rheumatism.

Natrum-muriaticum, on the contrary, has amelioration from perspiration. This is, indeed, Dr. Guernsey's great key-note for it.

Apis and Thuja have amelioration from perspiration in rheumatism.

Rhus-toxicodendron, Gelsemium, Cimex, and Ipecac. have amelioration from perspiration.

Although Hepar is given above as having perspiration without relief, yet Dr. Hering gives Hepar as having sore nodes on the head, which are relieved by perspiration.

Ferrum and Ipecac., according to Hering, have aggravation from perspiration. Ipecac. here furnishes a contradiction, like Hepar.

Mercurius has going to sleep of limbs when sitting quietly. In rheumatic conditions the patient can't lie in bed. As soon as he touches the bed he is worse.

The sleep of the Mercurius patient is easily disturbed, and he is addicted to walking about in his sleep at night.

Mercurius has irregular pulse, and so has its great antidote, Nitric-acid.

Mercurius is an unsafe remedy to give in typhoid fever.

This was one of Dr. Lippe's great hobbies. In twenty years of close association with him on the part of the editor, Dr.

Lippe was constantly protesting against the giving of Mercurius in typhoid fever, alleging that it insured fatal results. Once, when he lay sick in bed with a severe cold under the care of the editor, he fell to musing upon past experiences. He related a case of typhoid fever in which he was consulted by another physician. The symptoms seemed to point to Mercurius, and the other physician wished to prescribe it. Dr. Lippe protested, but without avail. The remedy was given by the attending physician, and not long afterward the patient died. Dr. Lippe used to say that Mercurius was *never* indicated in typhoid fever. Patients to whom it is given invariably die. To prescribe it in typhoid fever is simply to kill the patient. If the symptoms of a case seem to indicate it, do not give it, but examine the case more closely, and it will be found that some other remedy is better indicated.

No one who was not intimately acquainted with Dr. Lippe can form any idea of the positiveness of his convictions and of the earnestness and force with which he expressed them. The fierce intensity of his convictions of the unimpeachable truth of Homœopathy, the strength with which he defended it, and the violence of his denunciations of those who betrayed its principles, all constituted a phenomenon of strong belief that could be appreciated only by those who came in intimate association with this great apostle of Hahnemann.

Mercurius and Belladonna have chilliness on rising from bed.

During the fever Mercurius has much thirst for beer and other cold drinks.

Mercurius has perspiration toward morning, with palpitation. This is similar to Natrum-carbonicum and Nitric-acid. In this connection it would be well to remember the differential indications for Calcarea-carbonica and Silicea. Calcarea has perspiration in the first part of the night—during sleep—and Silicea has perspiration in the early morning.

Mercurius has caries of the bones, and so also have Calcarea-carbonica, Silicea, and Asafœtida.

Mercurius has eruptions, itching violently from the heat of the bed, bleeding when scratched.

Mercurius has moist itch, Sulphur dry itch, and Sepia army itch.

Mercurius has aggravation from walking in the open air. The Mercury patient inclines to lie on the back.

